# In the human sperm nucleus, nucleosomes form spatially restricted domains consistent with programmed nucleosome positioning

**DOI:** 10.1242/bio.041368

**Published:** 2019-07-01

**Authors:** Mei-Zi Zhang, Xiao-Min Cao, Feng-Qin Xu, Xiao-Wei Liang, Long-Long Fu, Bao Li, Wei-Guang Liu, Shuo-Guo Li, Fang-Zhen Sun, Xiu-Ying Huang, Wei-Hong Huang

**Affiliations:** 1 Reproductive Medicine Center, Tianjin First central hospital, Tianjin 300192, China; 2 Bejing Human Sperm Bank and National Research Institute for Family Planning, Beijing 100101, China; 3 Department of Urology, Affiliated Hospital of Weifang Medical University, 261000 Weifang, Shandong, China; 4 Center for Biological Imaging, Institute of Biophysics, Chinese Academy of Sciences, Beijing 100101, China; 5 State Key Laboratory of Molecular Developmental Biology, Institute of Genetics and Developmental Biology, Chinese Academy of Sciences, Beijing 100101, China

**Keywords:** Chromatin, Domain, Human sperm, Nucleosome

## Abstract

In human sperm, a fraction of its chromatin retains nucleosomes that are positioned on specific sequences containing genes and regulatory units essential for embryonic development. This nucleosome positioning (NP) feature provides an inherited epigenetic mark for sperm. However, it is not known whether there is a structural constraint for these nucleosomes and, if so, how they are localized in a three-dimensional (3D) context of the sperm nucleus. In this study, we examine the 3D organization of sperm chromatin and specifically determine its 3D localization of nucleosomes using structured illumination microscopy. A fraction of the sperm chromatin form nucleosome domains (NDs), visible as microscopic puncta ranging from 40 μm to 700 μm in diameter, and these NDs are precisely localized in the post acrosome region (PAR), outside the sperm's core chromatin. Further, NDs exist mainly in sperm from fertile men in a pilot survey with a small sample size. Together, this study uncovers a new spatially-restricted sub-nuclear structure containing NDs that are consistent with NPs of the sperm, which might represent a novel mark for healthy sperm in human.

## Abstract (precis)

**Summary:** Programmed gene expression is the key to life, confirmed experimentally in this study, which shows that nucleosomes in human sperm are specifically localized in genomic regions, guiding programmed gene expression.

## INTRODUCTION

In animal sperm, genomic DNA is mainly packaged by sperm nuclear basic proteins (SNBPs), which include protamines, protamine-like proteins and H1 histone-type proteins (Bloch and Teng, 1969; Ausió et al., 1999). In mammalian sperm, chromatin contains somatic histones to form nucleosomes and SNBPs to form nucleoprotamine. The retained nucleosomes package sequence-specific DNA fragments (Gatewood et al., 1987; Gardiner-Garden et al., 1998); whereas nucleoprotamine-containing chromatin forms the core chromatin. It is known that epsilon and gamma globin genes, which are expressed in the sperm, are embedded in nucleosomes in the sperm, but beta- and delta-globin genes, which are silent, are not associated with nucleosomes (Gardiner-Garden et al., 1998).

In mature human sperm, most of the retained nucleosomes are enriched in certain loci of the genome, such as imprinted genes and HOX genes, which are crucial for embryo development and can serve as epigenetic mark (Hammoud et al., 2009). This nucleosome-associated epigenetic information, such as histone H3 lys4 demethylation, in human and mouse sperm has been shown to be involved in spermatogenesis and cellular homeostasis, and has been used to mark developmental regulators by histone H3 Lys27 trimethylation (Brykczynska et al., 2010).

DNA sequences embedded in retained nucleosomes in human sperm have been mapped, which are closely related to the established DNA-methylation-free zone in the genomes of early embryos. From an evolutional point of view, the selective pressure on certain base compositions helps to retain specific nucleosomes, allowing successful transmission of specific paternal epigenetic information to the zygote (Vavouri and Lehner, 2011; Miller and Paradowska, 2013). In mouse sperm, only 1% of the genome is embedded into nucleosomes, and in human sperm the nucleosome-associated chromatin constitutes almost 15% of the human genome; retaining specific nucleosomes in the sperm as an epigenetic feature conserved over generations is consistent for both human and mouse (Erkek et al., 2013; Hisano et al., 2013).

Using proteomic mapping of human sperm, it has been found that retained nucleosomes constitute additional layers of epigenetic information (Castillo et al., 2014). Recent studies have shown that nucleosomes in human sperm are specifically positioned at transcription start sites (Erkek et al., 2013; Castillo et al., 2014). In genome-wide surveys of somatic cells and sperm, nucleosomes are preferentially positioned at internal exons, however this is independent of their epigenetic modification status, such as histone methylation and acetylation. This type of chromatin structure may involve general transcriptional regulation, exon recognition and splice-site selection (Nahkuri et al., 2009). However, certain conclusions about genome organization, based solely on computational analyses, has been challenged (Royo et al., 2016). New experiments to determine or validate structural features of the genome are needed.

While numerous studies suggest that nucleosome positioning in human sperm is important for early embryonic development and paternal epigenetic inheritance (Hammoud et al., 2009; Brykczynska et al., 2010; Vavouri and Lehner, 2011; Miller and Paradowska, 2013), specific nucleosome positioning has yet to be experimentally confirmed. Further, the information regarding any 3D localization of nucleosome domains is entirely unknown. In this study, we determine the 3D positioning of nucleosomes in human sperm by using super-resolution structured illumination microscopy (SIM). We conclude that in human sperm, histone-containing chromatin forms nucleosome domains (NDs), which are specifically localized at PAR and represent a structural feature of chromatin organization in the sperm. Further, NDs were mainly found in the sperm from fertile men in a pilot survey, indicating that NDs might be associated with a healthy status of sperm in human.

## RESULTS

We first obtained healthy sperm samples for this study; the quality of sperms used in the study is shown in Table 1. Based on WHO morphology guidelines, the fresh semen sample contained a high percentage (68%) of sperm exhibiting normal morphology, indicative of fertility. After gradient centrifugation through PureSperm 100 media, the purified sperm fraction had a 95% motility rate, a 98% progression and a 96% normal morphology.

**Table 1.**
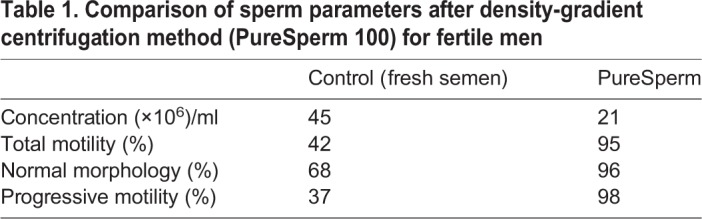
**Comparison of sperm parameters after density-gradient centrifugation method (PureSperm 100) for fertile men**

**Fig. 1.**
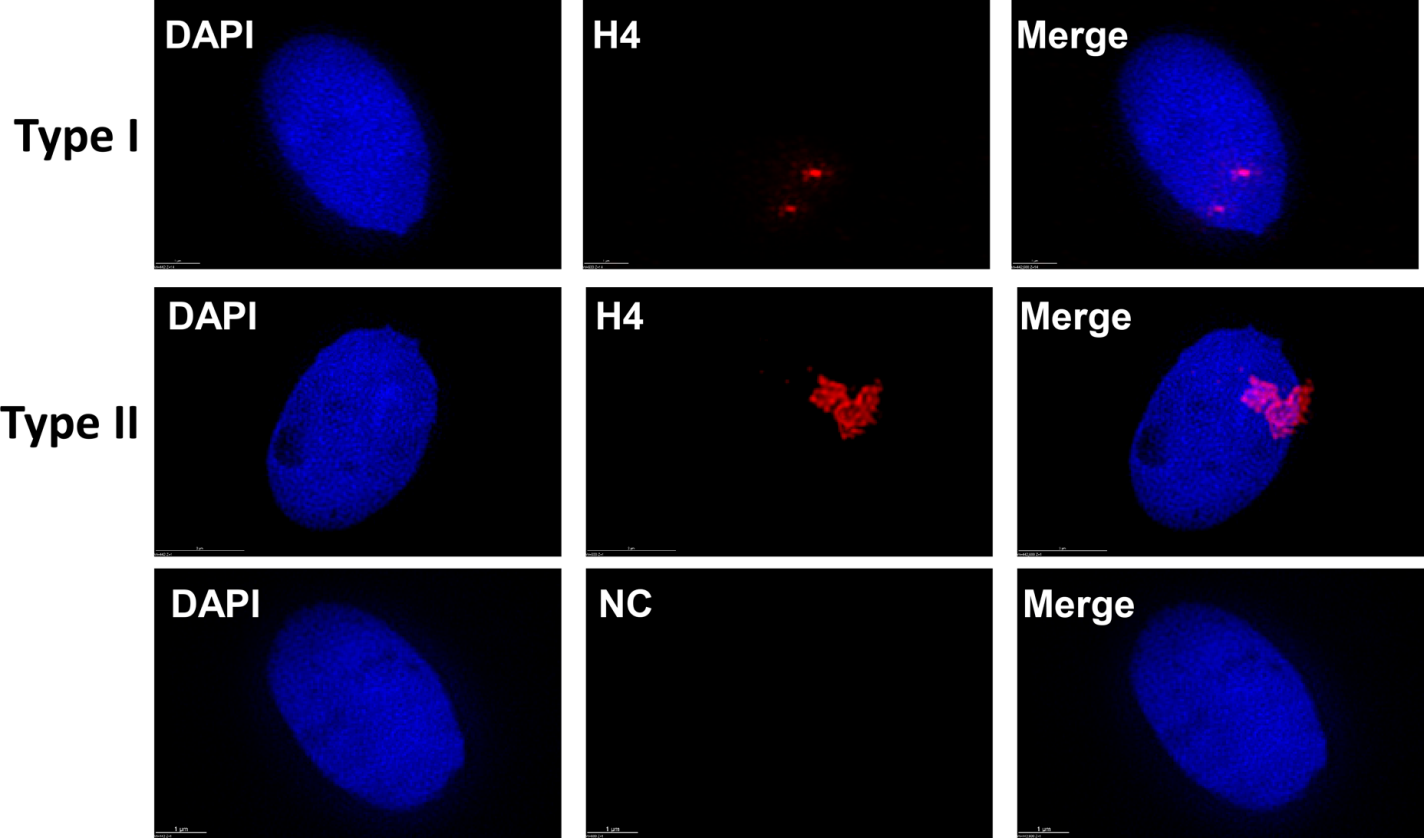
**Nucleosomes in human sperm localize in PAR with sparse or aggregated dots to form NDs.** (Top row) Type I NDs with sparse dots. The diameter of NDs ranges from 40–700 μm. (Middle row) Type II NDs with aggregated dots. Type I is major, while type II is minor. (Bottom row) Negative controls were performed in the absence of primary antibody. H4, histone H4; NC, negative control. Scale bars: (upper and lower rows) 1 µm, (middle row) 2 µm.

We embedded purified sperm in a 20% gelatin solution, and then in optimal cutting temperature compound (OCT compound), which maintains the 3D structure of the sperm, allowing 3D structures to be determined by SIM microscope. Cryosections of the OCT-embedded sperm block were obtained at a thickness of 4 μm, which were subjected to immunostaining using histone H4 antibody alone or in conjunction with protamine 1 antibody. The slides were examined by SIM and images were analyzed by softWoRx Suite software (GE, USA). Histone H4-positive signals indicated the presence of nucleosomes. The staining patterns could be classified into two major types, with type I containing sparse puncta of H4 signals and type II containing aggregated puncta of H4 signals (Fig. 1A,B, Movies 1 and 2). These distinct puncta indicated that nucleosomes in human sperm form specific structures, such as NDs, because the size of these puncta are much larger than that of a single nucleosome (around 10 nm). Type I structures represented the dominant type, whereas type II was only occasionally seen. These types of histone H4-positive NDs were found in most of the sperms, which were also found by using histone H2B antibody (Fig. S1, Movie 3). The statistical results are shown in Table 2.

**Table 2.**
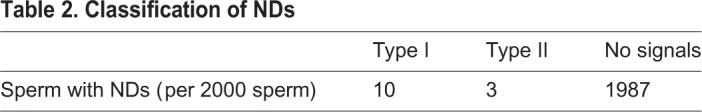
**Classification of NDs**

**Fig. 2.**
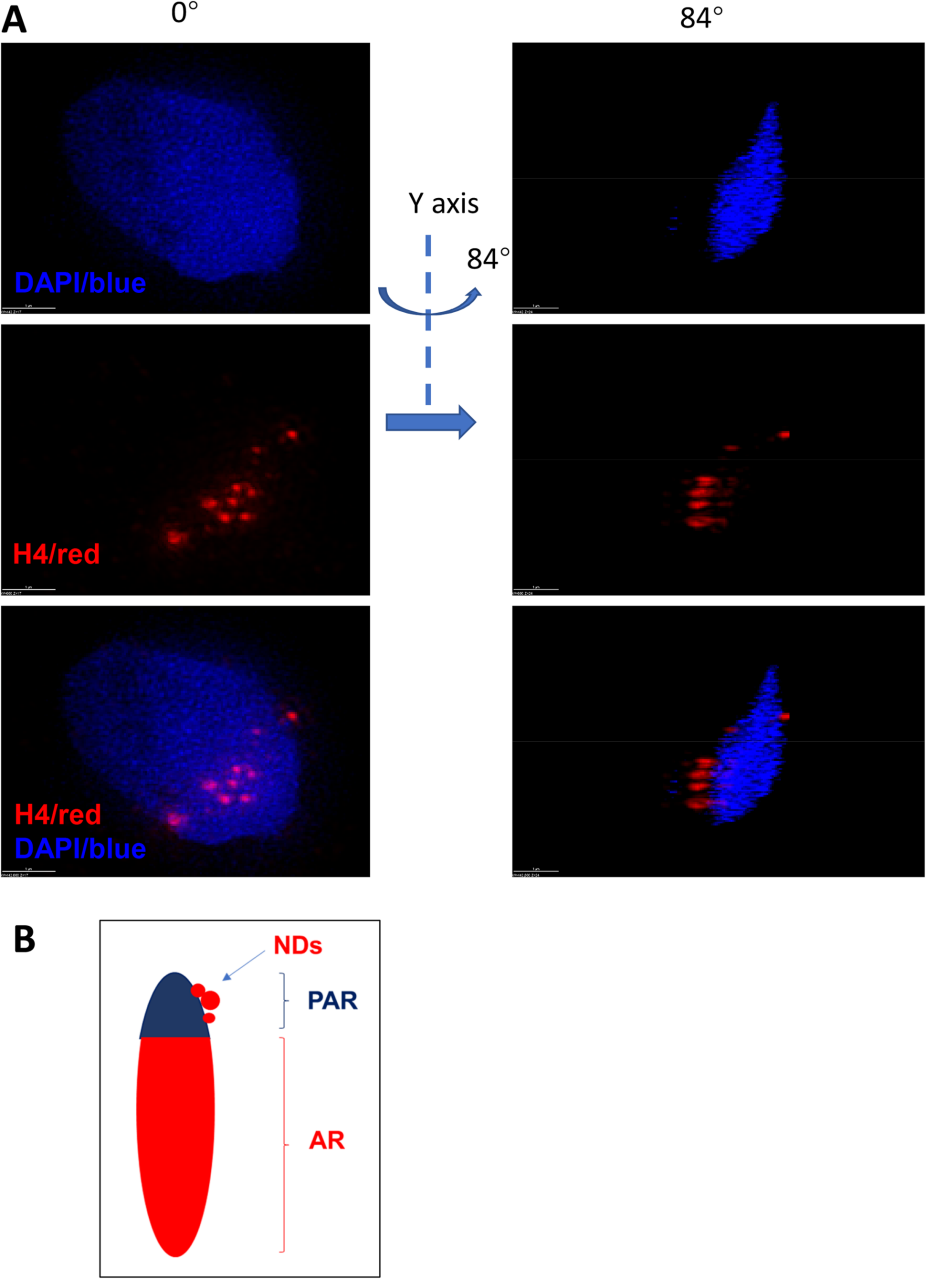
**Using SIM the 3D positioning of NDs is shown.** (A) After rotation around the Y-axis, NDs were shown to localize outside the core chromatin in PAR. The NDs were not shown to be inside of sperm chromatin. (B) A diagram of NDs positioning outside the major dense sperm chromatin. Scale bars: 1 µm.

Interestingly, NDs were strictly localized around the PAR, and were outside of the zone of core chromatin (Fig. 2 and Fig. S2, Movie 4). The size of NDs ranged from 40 to 700 μm in diameter. The number of NDs in a sperm ranged from 9 to 67, but the major NDs only ranged from 1 to 19 (Table 3).

**Table 3.**
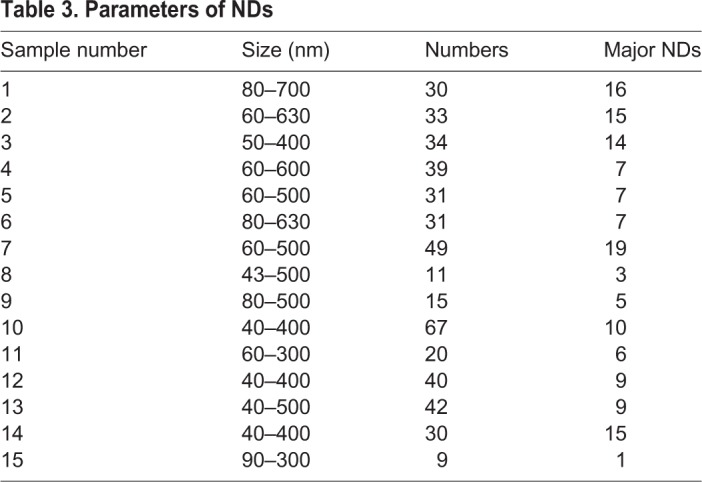
**Parameters of NDs**

**Fig. 3.**
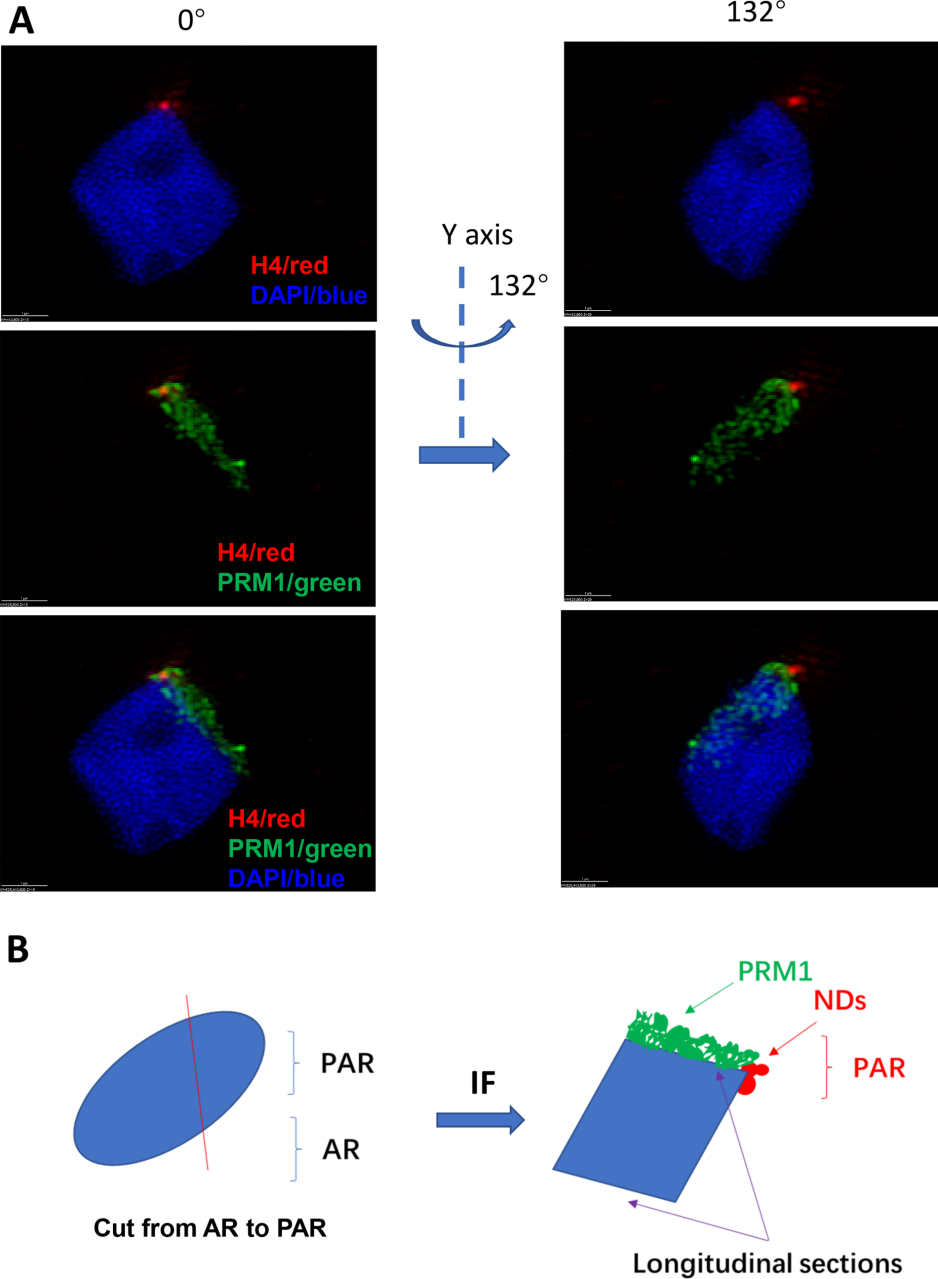
**After cutting through AR and PAR, localization of NDs is shown.** (A) After sperm was cut on both sides, the inner side stuck to the slide without antibody staining, but the outer side was stained by both H4 and protamine 1 antibodies. H4 signals were confined in the PAR and outside of the inner chromatin, but not in the inner chromatin. However, protamine 1 signals ranged from the AR to the PAR in inner chromatin in the longitude section. (B) A diagram showing that NDs are not localized in the inner chromatin, but outside of the sperm chromatin in the PAR. Scale bars: 1 µm.

**Fig. 4.**
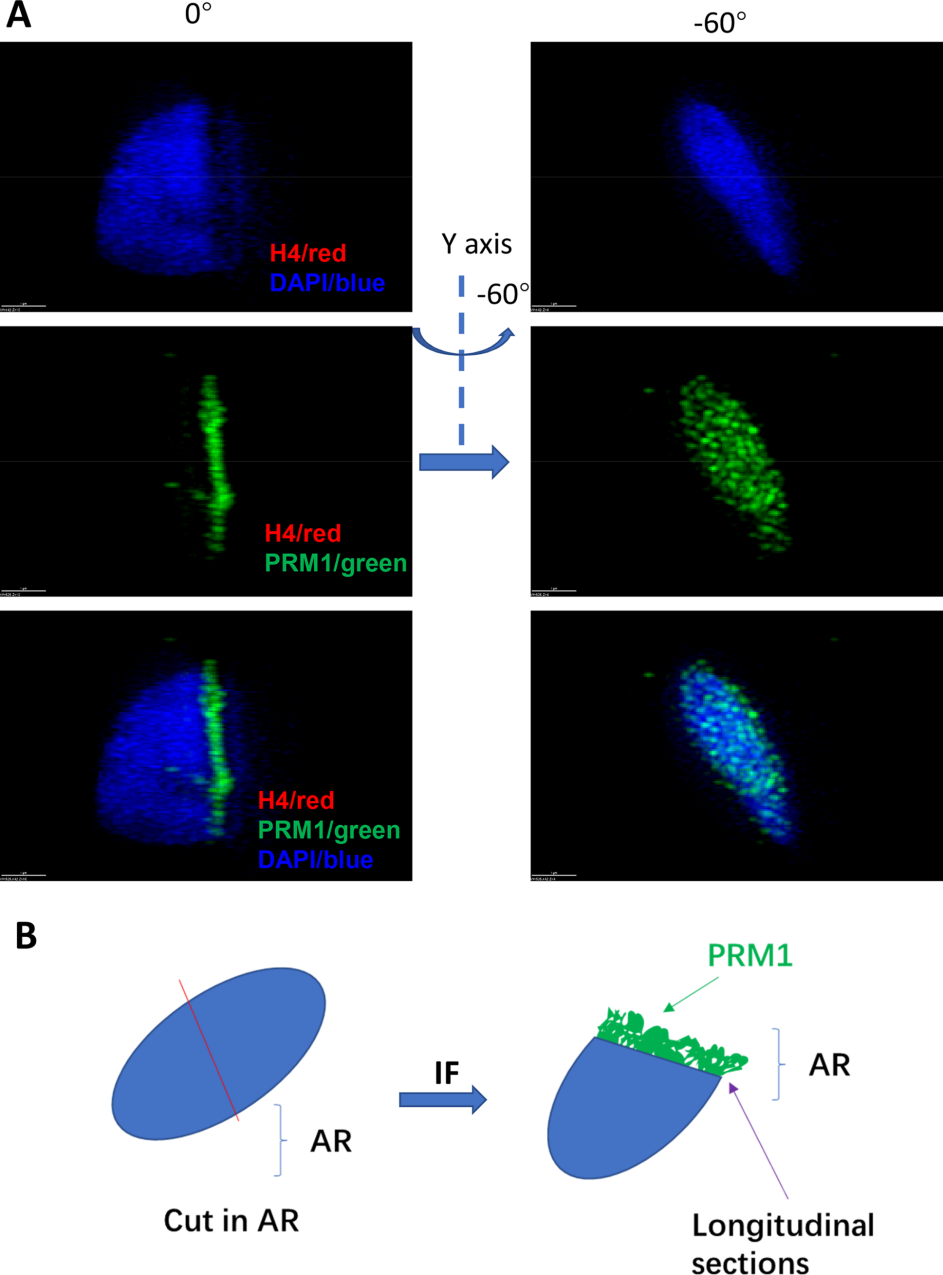
**After cutting through AR, NDs are not detected.** (A) Sperm was cut in AR. After staining by both H4 and protamine 1 antibodies, H4 signals were not detected, while protamine 1 signal was full in the inner chromatin in the longitude section. (B) A diagram showing that NDs are not detected in the inner and outer sides of sperm chromatin in AR. Scale bars: 1 µm.

To further localize the NDs in sperm chromatin, we examined those sections spanning the sperm inner chromatin, and determined whether NDs were also localized in the inner sperm chromatin. Protamine 1 stained the sperm core chromatin. On longitudinal sections cut through the acrosome region (AR) or PAR, we found that those sections spanning the AR were protamine 1-positive and H4-negative. However, those sections spanning the PAR were H4-positive only outside the core sperm chromatin (Figs 3, 4, and Fig. S3, Movies 5–8).

These data show that nucleosomes in human sperm form NDs which are specifically localized, proximal to the PAR. This is consistent with studies suggesting that nucleosomes in human sperm form on certain specific sequences of the genome (Hammoud et al., 2009; Gatewood et al., 1987, 1990). It has been proposed that specific nucleosome positioning (NP) in the human sperm genome is important in guiding programmed gene expression in early embryonic development and may represent a form of epigenetic inherence (Hammoud et al., 2009; Vavouri and Lehner, 2011). We thus propose that NDs observed in our study may reflect NP in the sperm. Further, we collected six infertile samples and six fertile samples and surveyed the distribution of NDs in these samples. Interestingly, none of the six infertile samples exhibited any form of ND, but all six fertile samples contained sperm with NDs (Table 4). The results imply that NDs in human sperm may be relative to the health status of the sperm.

**Table 4.**
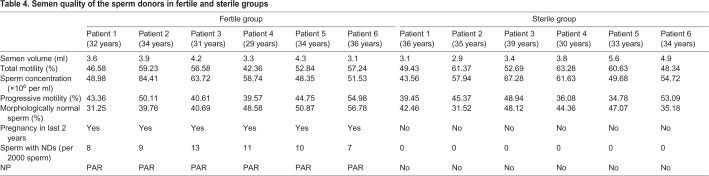
**Semen quality of the sperm donors in fertile and sterile groups**

Taken together, this study indicates that NDs, which are specifically localized and proximal to the PAR, could represent a new type of 3D structural feature in human sperm. Further, in a pilot survey the presence of NDs is shown to be mainly in fertile sperm, indicating that NDs may be relative to the health status of sperm in human.

## DISCUSSION

Though where nucleosomes are localized in the mammalian sperm genome remains controversial, it is a generally consistent view that retained nucleosomes in the sperm influence embryonic development and epigenetic inheritance (Gatewood et al., 1987; Hammoud et al., 2009; Carone et al., 2014; Samans et al., 2014).

It is hypothesized that sequence-specific NP facilitates sperm chromatin decondensation (SCD) upon fertilization, and that the position represents the start site of SCD (Gatewood et al., 1987). SCD occurs immediately after membrane fusion of gametes (Ajduk et al., 2006) and the site in the sperm for membrane fusion is also the start site of SCD, which has been shown to be around the PAR (Yanagimachi and Noda, 1970; Fléchon and Pavlok, 1986). To confirm this result, we used FITC-PNA to detect acrosome and histone H4 to detect NDs. The result showed that NDs were not overlapped with acrosome region, but localized in the PAR (Fig. S4). In this study, we present evidence for NDs in the sperm, and show that NDs form around the PAR.

Previous studies have shown that nucleosomes in human sperm package specific DNA sequences, which provide epigenetic marks and are required for embryonic development (Gatewood et al., 1987; Hammoud et al., 2009; Brykczynska et al., 2010). In this study, our data demonstrate that in human sperm, nucleosomes form distinct NDs which are specifically localized to where NP is suggested to exist. This would indicate that both NDs and NP in the sperm are the results of important, programmed events during spermatogenesis, and support embryonic development after fertilization.

Strikingly, NDs are not found in inner chromatin. As protamines in core chromatin might handle the binding of histone antibodies and previous studies had showed that without SCD immunostaining for protamines and histones in human and mouse sperm was failed (van der Heijden et al., 2006; Govin et al., 2007), single nucleosomes might still sparsely distribute in inner chromatin. To confirm this result, we detected nucleosomes in inner chromatin after sperm chromatin decondensation. The result showed that inner chromatin contains abundant nucleosomes (Fig. S5). However, as NDs are large units and unlikely to be fully inhibited by protamines during immunostaining, it is very likely that NDs are not localized in inner chromatin.

As nucleosomes of mammalian sperm will transmit into zygote (van der Heijden et al., 2006), paternal epigenetic information on NP should be programmed and transferred to their children. In previous studies (Carone et al., 2014; Gatewood et al., 1990), when MNase was used to digest human sperm chromatin, dinucleosomal bands and nucleosome ladders were found. The researchers then hypothesized that nucleosome retention occurs in blocks with adjacent nucleosomes. Our data are highly consistent with those observations as NDs are programmed to precisely aggregate in the PAR and outside of inner chromatin.

In previous studies, CHIP-seq has been used for identifying the regions covered by retained nucleosomes in the sperm genome (Carone et al., 2014; Hammoud et al., 2009; Samans et al., 2014; Teperek et al., 2016; Yamaguchi et al., 2018). Nucleosomes without significant histone modifications are predominantly localized at gene deserts. H3K9me3-containing nucleosomes are enriched in satellite repeats. H3K4me3-containing nucleosomes are highly enriched at high CpG promoters and in TSSs, which seems to be a universal phenomenon in individual sperm (Yamaguchi et al., 2018). In consistency with those studies, H3K36me3 was detected in NDs, but H3K4me3 was not detected in NDs (Fig. S6). These data indicate that retained nucleosomes in sperm genome play important regulatory roles.

After fertilization, nucleosomes in mammalian sperm involved in hyperacetylation and transcription activation, in which paternal chromatin is more active than maternal chromatin before activation of zygotic genome (ZGA) (Adenot et al., 1997; Thompson et al., 1995). The nucleosome retained in human sperm then could quickly and readily meet the requirement for zygotic development before ZGA.

Infertility affects around 15% couples around the world, and male infertility accounts for 40–50% of it (Eliasson, 2010; Menkveld et al., 2011; Mackenna, 1995; Zayed et al., 1997). In this study, our data indicate that NDs and NP might constitute additional layers of epigenetic information in human sperm, and exist mainly in sperm from fertile men, which then could be a new target in diagnosing male infertility in terms of possible defects of NDs in sterile men.

It is well known that expression of imprinting paternal genes from infertile men is dysregulated during early embryonic development (Botezatu et al., 2014; Hiura et al., 2014; Kobayashi et al., 2007, 2017; Laurentino et al., 2015). Paternal X chromosome is active on transcription of X-linked genes after fertilization (Patrat et al., 2009), which raises the possibility that X-linked genes might rely on the retained nucleosomes in sperm for supporting their quick and efficient expression. Therefore, it is important to test if the regions covered by nucleosomes in the sperm genome are enriched with imprinting genes and X-linked genes in future.

## MATERIALS AND METHODS

### Specimen collection

All procedures were in accordance with institutional guidelines on Human Subjects in Experimentation of the Institute of Genetics and Developmental Biology (CAS, China), Tianjin First Central Hospital, and National Research Institute for Family Planning. Semen samples were collected by manual masturbation from consenting men with known fertility (having a child within the past 2 years) or with known sterility (without a child in history by natural conception, but the semen parameters are normal. Their partner had not conceived in IUI, IVF and ICSI using their semen, but had conceived in IUI by use of healthy donor's semen).

### Specimen processing

After liquefaction at 37°C for 60 min in an air incubator with 5% CO_2_, seminal plasma was removed by centrifugation at 300 ***g*** for 10 min, through Enhance S-Plus Cell Isolation Media (mHTF, Vitrolife, https://www.vitrolife.com). The cells were washed in Modified Human Tubal Fluid medium (Irvine Scientific, www.irvinesci.com) once, and pelleted by centrifugation at 700 ***g*** for 10 min. The sperm were resuspended in mHTF at a high concentration (about 200×10^6^ per ml) fixed by 4% paraformaldehyde (pH 7.4) in 1× PBS for overnight at 4°C. After removing excess paraformaldehyde with PBS washing, the fixed sperm was subjected for gradient sucrose infiltration. Further, after removal of excess sucrose from the fixed sperm, it was embedded in OCT compound. After the fixed sperm was frozen, it was placed into plastic bags, sealed and stored at −20°C.

### PureSperm density gradient centrifugation

By using a PureSperm 100 discontinuous density gradient (Nidacon, Gothenburg, Sweden), a two-layer gradient was prepared by dilution solutions of 40% and 80% PureSperm. After the whole media was pre-warmed to 37°C, liquefied semen sample was placed on top of the upper layer and centrifuged at 300 ***g*** for 20 min following the product's instructions. The pellet was washed twice by wash solutions and recovered; aliquots were used for storage at liquid nitrogen or used for a routine semen analysis according to the World Health Organization guidelines (2010, 5^th^ ed.).

### Cryopreservation of spermatozoa and thawing

After purified sperm was diluted in Sperm Maintenance Medium (SMM, Irvine Scientific, USA) at the ratio of 3:1, the mixture was transferred to 0.5 ml straw (PETG sperm 0.5 ml, clear straw, Irvine Scientific, CA, USA). That was suspended for 40 min in liquid nitrogen vapor, and then plunged into liquid nitrogen for long storage.

After the straw was pulled out from liquid nitrogen, it was immersed immediately into pre-warmed mineral oil at 38°C. After warming, sealer of the straw was removed, and sperm was washed once by Sperm Washing Medium (Irvine Scientific, CA, USA).

### Decondensation of human spermatozoa

After semen from a fertile man was washed by Krebs Ringer bicarbonate medium buffered with Hepes (KRB-Hepes, 119.4 mM NaCl, 4.8 mM KCl, 1.7 mM CaCl_2_, 1.2 mM KH_2_PO_4_, 1.2 mM Mg_2_SO_4_, 4 mM NaHCO_3_, 21 mM Hepes, 1 mM sodium pyruvate, 25 mM sodium lactate, 5.6 mM glucose, 1 U/ml of penicillin G and 1 mg/ml of streptomycin sulfate at pH 7.4) three times, sperm smears were prepared on pre-washed glass slides. The smears were briefly fixed in 4% paraformaldehyde for less than 2 min and air-dried. Then, smears were incubated with KRB-Hepes buffer for 30 min to be rehydrated. Further, a decondensation mixture consisting of 10 mM dithiothreitol and 10 μg/ml heparin was incubated with the smears for 60 min. After the smears were washed by KRB-Hepes three times, the smears were subjected for immunocytochemistry.

### Immunohistochemistry

After thawing, sperm was fixed in 4% paraformaldehyde in phosphate buffered saline (1× PBS; pH 7.4) for 30 min at room temperature or overnight at 4°C. The fixed sperm was centrifuged at 3000 ***g*** for 5 min, and the pellet was embedded into 20% gelatin in water at 42°C. After the samples were solidified at 4°C for 1 h, they were embedded in OCT compound (Thermo Fisher Scientific) and frozen in liquid nitrogen. Frozen blocks of sperm were then cytosectioned at 4 µm thickness (Leica CM3050 S, Leica, Germany), and the slices were put on slides and dried at room temperature for at least 2 h. The slides were further washed by PBS with 0.1% tween-20 and 0.01%Tx-100, and they were permeabilized with 0.5% Triton X-100 in PBS at room temperature for 1 h. After being washed by PBS with 1% bovine serum albumin (BSA) twice, they were blocked in 1% BSA -supplemented PBS in for 1 h at RT. After incubation overnight at 4°C in the appropriate antibodies [Histone-H4 Rabbit Polyclonal antibody (1:100, Proteintech Group, 16047-1-AP), Histone-H2B Rabbit Polyclonal antibody (1:100, Proteintech Group, 15857-1-AP), Histone-H3 Rabbit Polyclonal Antibody (1:100, Proteintech Group, 17168-1-AP), Histone H3 (tri methyl K4) Rabbit polyclonal antibody (1:150, Abcam, ab8580), Histone H3 (tri methyl K36) Rabbit polyclonal antibody (1:150, Abcam, ab9050), or Protamine 1 Antibody (A-17) (1:200, Santa Cruz, sc-23107)] diluted in 1% BSA-supplemented PBS, they were washed in PBS containing 0.1% Tween 20 and 0.01% Triton X-100 for 5 min each three times. Then, a secondary antibody, labeled with Alexa Fluor 594 or 488 (Goat Anti-Rabbit IgG-Alexa Fluor 594, ab150080; Goat Anti-Rabbit IgG-Alexa Fluor 488, ab150077; Donkey Anti-Goat IgG-Alexa Fluor 488, ab150129), was used as the fluorescent reporter for the primary antibodies. Then they were post-fixed with 4% formaldehyde in PBS for 30 min at RT. After washing for 5 min by PBS with 0.1% tween-20 and 0.01% Tx-100 three times, they were stained by DAPI (1 μg/ml) for 20 min at RT. After washing by PBS containing 0.1% Tween 20 and 0.01% Triton X-100 for 5 min each twice, they were drained and mounted in ProLong Gold Antifade Reagent (Life Tech). The sealed slides were used for 3D study by SIM.

### 3D-SIM super-resolution microscopy and image analysis

SIM was performed on the DeltaVision OMX V3 imaging system (Applied Precision) with Plan Apochromat 100×/1.46 oil objective, 1.6× lens in the detection light path and Andor iXon 885 EMCCD camera; Z-stacks were acquired with 125 nm intervals. In three-color images, channels (405, 488 and 593 nm) were acquired sequentially using standard single-band filter sets. SIM image stacks and raw SIM images were analyzed by softWoRx 5.0 (Applied Precision). Further, reconstructed images were rendered in three dimensions also by softWoRx 5.0 and linear adjustments to brightness were performed on 3D reconstructions for better contrast.

### Quantification of nucleosome domains and positions

The nucleosome domains and positions were analyzed by softWoRx 5.0 software. The measurement module was used to calculate the size of NDs. Briefly, using the ‘measure tool’, the method is ‘multiple segment’ and the unit is ‘micrometers’. Manually selecting a start point and an end point on one ND, the software will return the length between the two points. This length was then considered to be the diameter of the ND.

## Supplementary Material

Supplementary information
