# Supplementary material for: In the human sperm nucleus, nucleosomes form spatially restricted domains consistent with programmed nucleosome positioning
Source: Biol Open. 2019 Jul 1;8(7):bio041368. doi: 10.1242/bio.041368 (PMC6679404; doi:10.1242/bio.041368)
Supplement: Supplementary information [file biolopen-8-041368-s1.pdf]

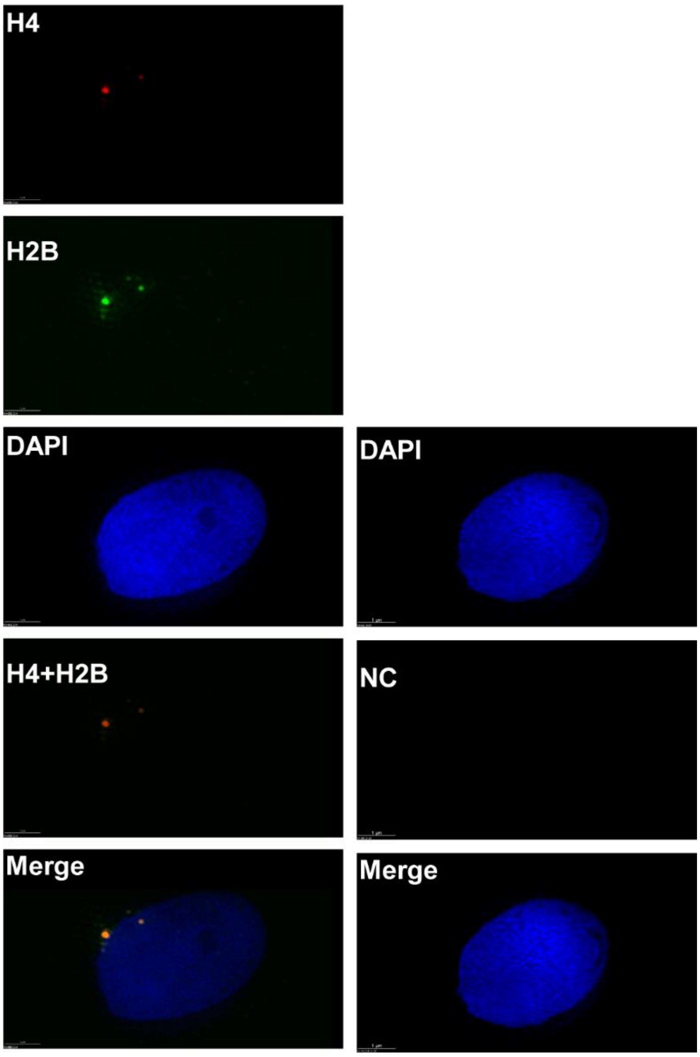

Figure S1. NDs were also detected by use of histone H2B antibody

Similar results were obtained by use of histone H2B antibody. NDs with sparse dots were shown outside of sperm chromatin in PAR. Negative controls were performed in the absence of primary antibody.

Biology Open • Supplementary information

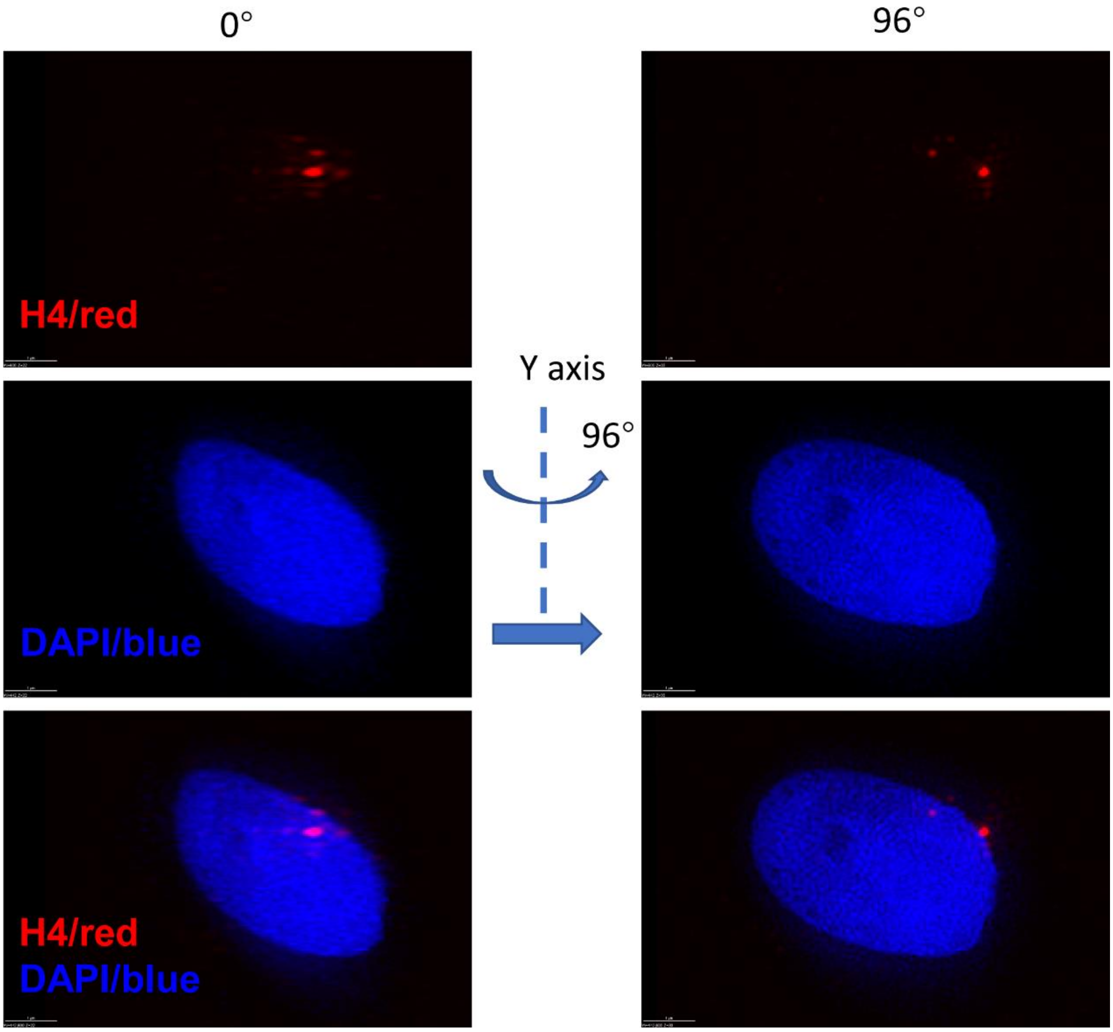

Figure S2. NDs was shown to be exactly localize outside of core chromatin in PAR. Similar results were obtained in another sample. NDs with sparse dots were shown outside of inner chromatin in PAR. Negative controls were performed in the absence of primary antibody. This data is similar to Figure 2.

Biology Open • Supplementary information

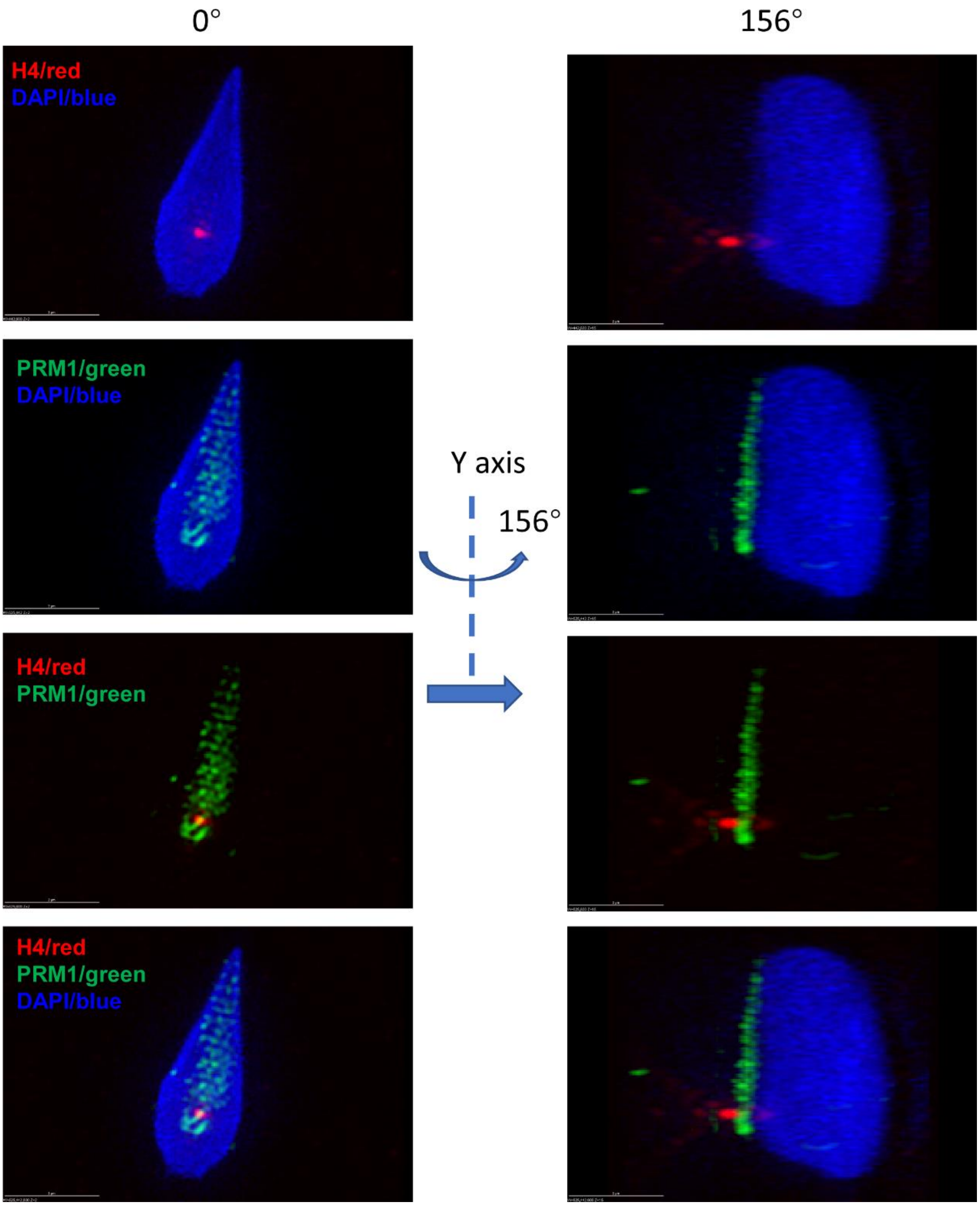

Figure S3. NDs was confined in the PAR and outside of inner chromatin, but not in inner chromatin.

Similar results were obtained in another sample to show that NDs localized outside of sperm chromatin but not in the inner sperm chromatin. NDs with sparse dots were shown

Biology Open • Supplementary information

outside of inner chromatin in PAR. Negative controls were performed in the absence of primary antibody. This data is similar to Figure 3.

Biology Open • Supplementary information

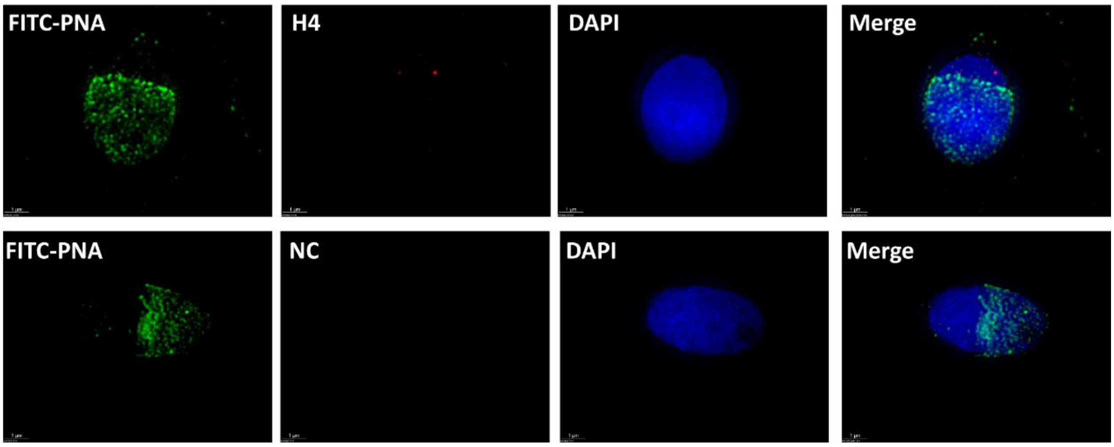

Figure S4. Localization of histone H4 (red) on human spermatozoa

The sperm smears stained with FITC-PNA and antibody against histone H4 following by a Coralite 594 conjugated donkey anti-rabbit IgG (1:1000 dilution); sperm nuclei stained by DAPI (light blue); in NC (Negative control) group, sperm stained by the secondary antibody alone; acrosome stained by FITC-PNA .

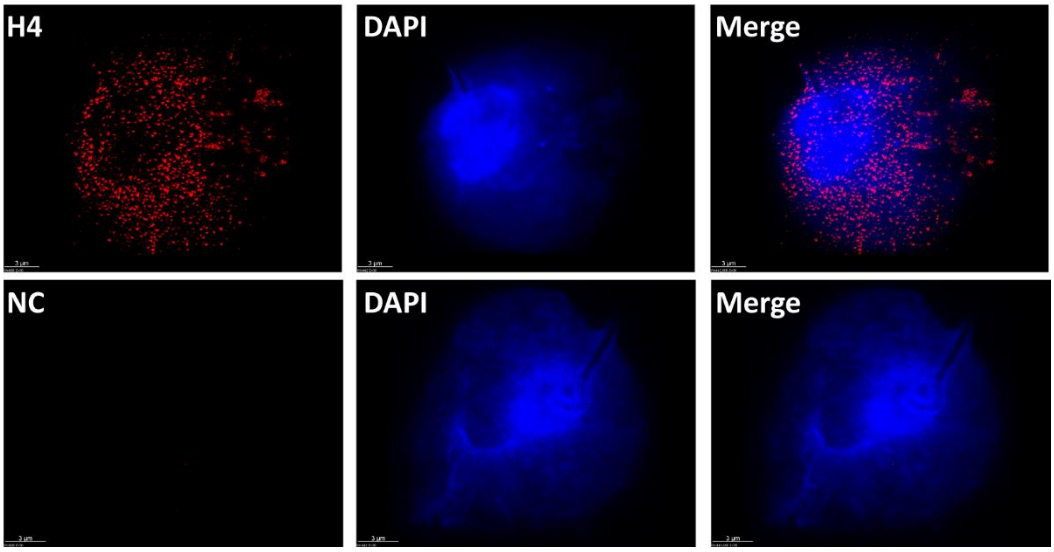

Figure S5. Localization of histone H4 on decondensed human spermatozoa

The decondensed sperm stained with antibody against histone H4 following by a Coralite 594 conjugated donkey anti-rabbit IgG (1:1000 dilution); sperm nuclei stained by DAPI (light blue); in NC (Negative control) group, sperm stained by the secondary antibody alone.

Biology Open • Supplementary information

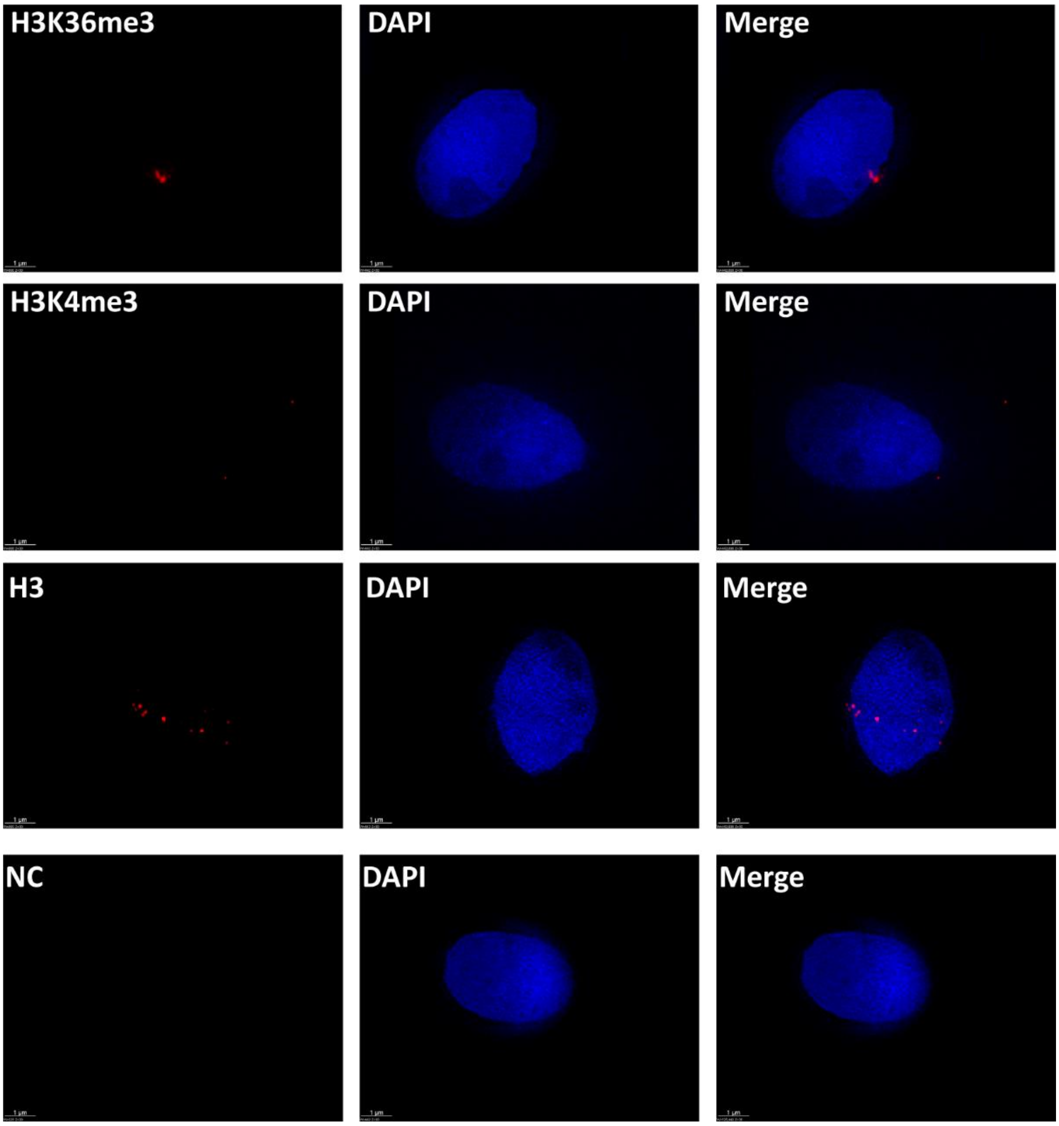

Figure S6. Localization of histone H3K36me3, H3K4me3 and H3 on human spermatozoa

The sperm smears stained with antibody against histone H3, H3K4me3, and H3K36me3 following by a Coralite 594 conjugated donkey anti-rabbit IgG (1:1000 dilution); sperm nuclei stained by DAPI (light blue); in NC (Negative control) group, sperm stained by the secondary antibody alone.

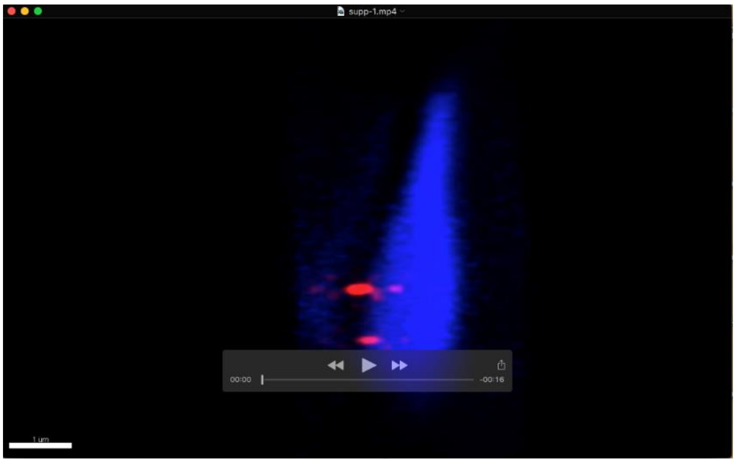

Movie 1. SIM movie showing type I of nucleosomal domains. Sparse puncta of histone H4 signals (red) and nucleus stained by DAPI (blue) are shown. Related to Figure 1A.

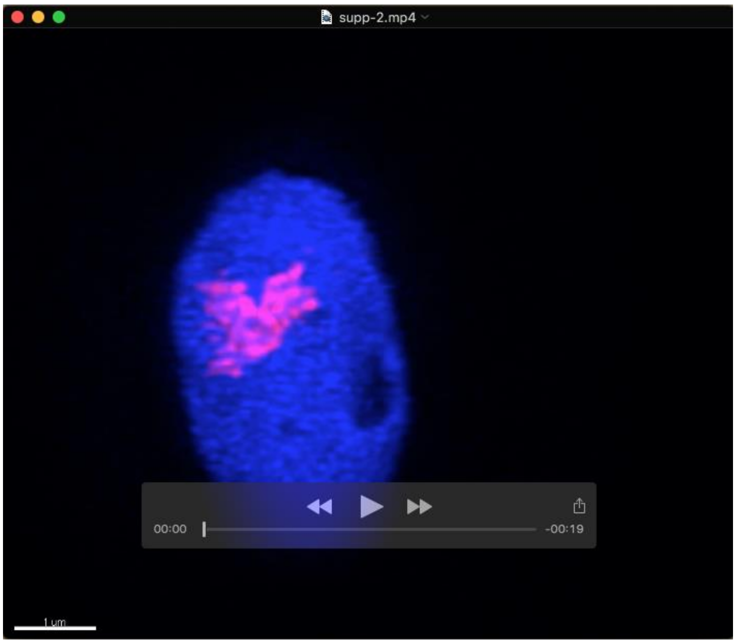

Movie 2. SIM movie showing type II of nucleosomal domains. Aggregated puncta of histone H4 signals (red) and nucleus stained by DAPI (blue) are shown. Related to Figure 1B.

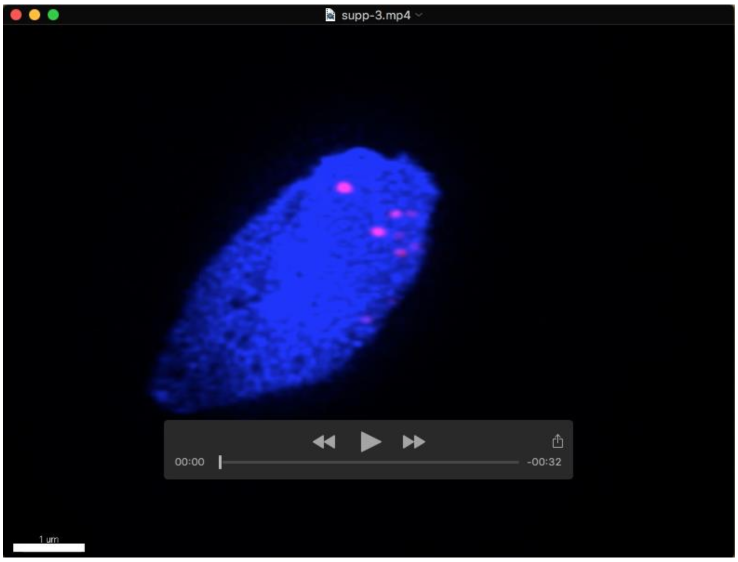

Movie 3. SIM movie showing that NDs are shown to localize in PAR. Histone H4 signals (red) and nucleus stained by DAPI (blue) are shown. Related to Figure 2.

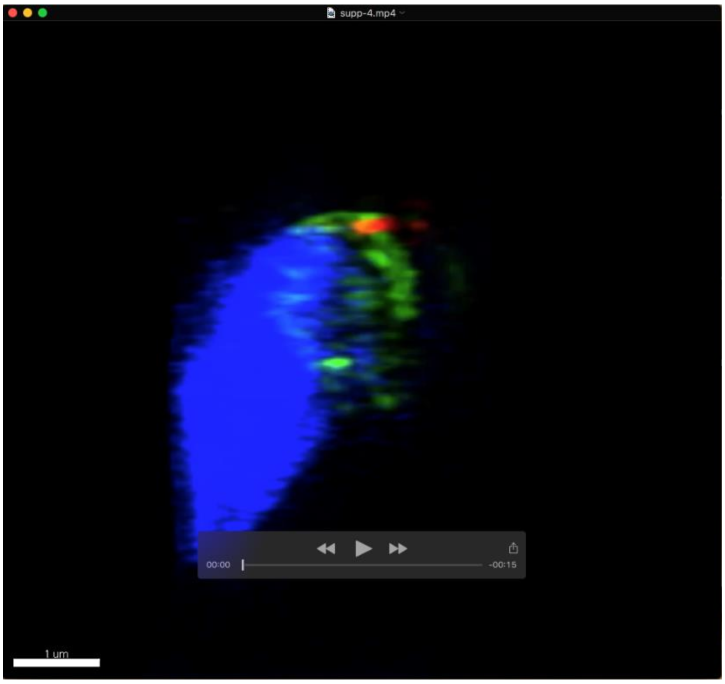

Movie 4. SIM movie showing that NDs are positioning outside of major dense sperm chromatin. Histone H4 signals (red), protamine 1 signals (green), and nucleus stained by DAPI (blue) are shown. Related to Figure 3.

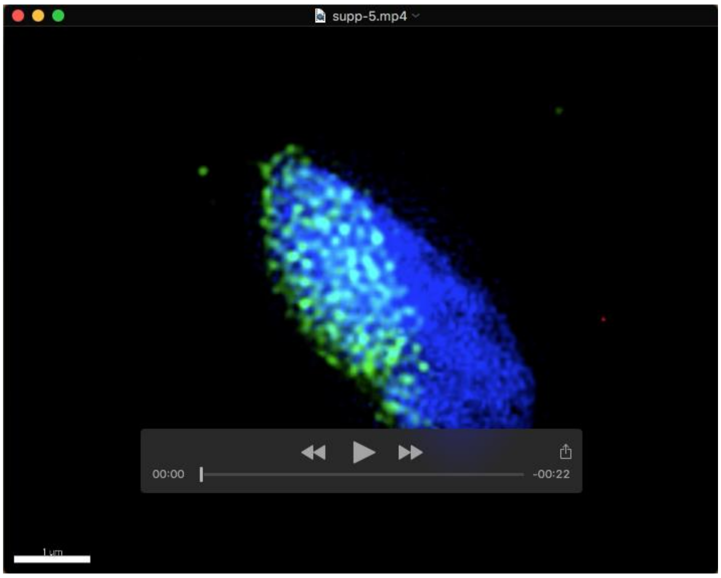

Movie 5. SIM movie showing that NDs are not found in the inner sperm chromatin in AR. Histone H4 signals (red), protamine 1 signals (green), and nucleus stained by DAPI (blue) are shown. Related to Figure 4.

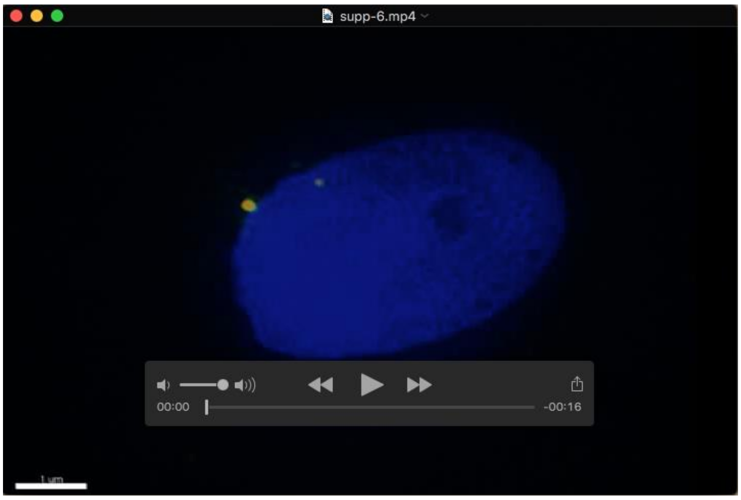

Movie 6. SIM movie showing that NDs are also detected by use of histone H2B antibody. Histone H4 signals (red), histone H2B signals (green), and nucleus stained by DAPI (blue) are shown. Related to Figure S1.

Biology Open • Supplementary information

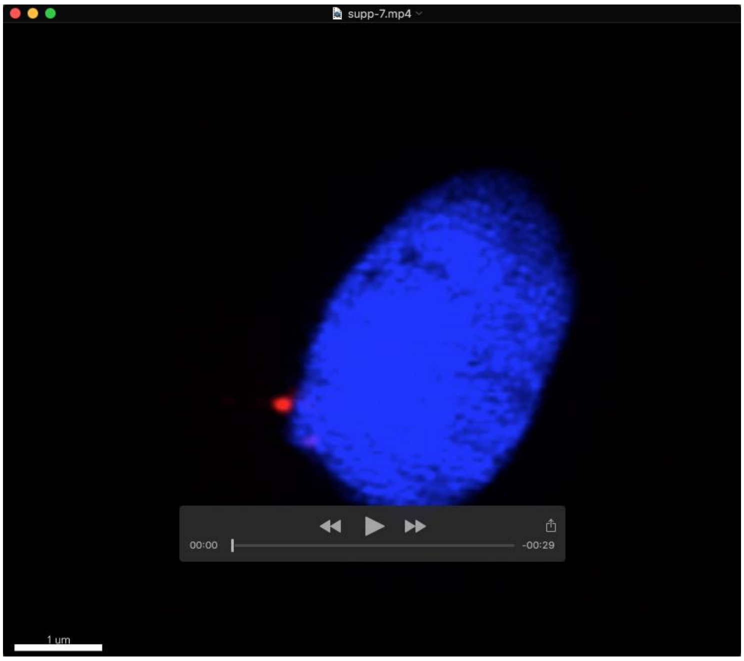

Movie 7. SIM movie showing that NDs are shown to localize in PAR in another similar result. Histone H4 signals (red) and nucleus stained by DAPI (blue) are shown. Related to Figure S2.

Biology Open • Supplementary information

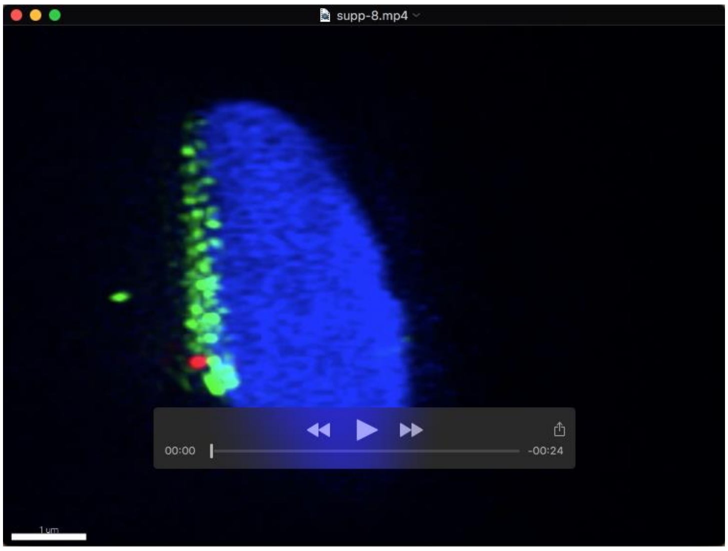

Movie 8. SIM movie showing that NDs are positioning outside of major dense sperm chromatin in another similar result. Histone H4 signals (red), protamine 1 signals (green), and nucleus stained by DAPI (blue) are shown. Related to Figure S3.

Biology Open • Supplementary information
